# Targeting Cognitive Resilience through Prebiotics: A Focused Perspective

**DOI:** 10.1016/j.advnut.2024.100343

**Published:** 2024-11-16

**Authors:** Boushra Dalile, Neil B Boyle, Franco T Ruiz, Anirikh Chakrabarti, Frederique Respondek, Georgina F Dodd, Kathrin Cohen Kadosh, Piril Hepsomali, Robert J Brummer, Simon McArthur, Veerle Dam, Yoghatama Cindya Zanzer, Yannick Vermeiren, Harriet Schellekens

**Affiliations:** 1 Brain Research on Affective Mechanisms (BRAMLab), Laboratory of Biological Psychology, Research Unit Brain & Cognition, Faculty of Psychology and Educational Sciences, KU Leuven, Leuven, Belgium; 2 School of Psychology, University of Leeds, Leeds, United Kingdom; 3 Department of Psychology, University of Sheffield, Sheffield, United Kingdom; 4 Translational Research Center for Gastrointestinal Disorder (TARGID), Department of Chronic Diseases and Metabolism, Faculty of Medicine, KU Leuven, Leuven, Belgium; 5 Cargill Inc., Vilvoorde, Belgium; 6 CP Kelco, Levallois-Perret, France; 7 Clasado Biosciences, Reading, United Kingdom; 8 School of Psychology, Faculty of Health and Medical Sciences, University of Surrey, Guildford, United Kingdom; 9 School of Psychology and Clinical Language Sciences, University of Reading, Reading, United Kingdom; 10 School of Medical Sciences, Faculty of Medicine and Health, Örebro University, Örebro, Sweden; 11 Institute of Dentistry, Faculty of Medicine & Dentistry, Queen Mary University of London, London, United Kingdom; 12 Sensus B.V., Roosendaal, The Netherlands; 13 BENEO Institute/Südzucker Group, Obrigheim/Pfalz, Germany; 14 Division of Human Nutrition and Health, Chair Group Nutritional Biology, Wageningen University & Research (WUR), Wageningen, The Netherlands; 15 APC Microbiome Ireland, University College Cork, Cork, Ireland; 16 Department of Anatomy and Neuroscience, University College Cork, Cork, Ireland

**Keywords:** prebiotics, cognitive resilience, cognition, gut-brain axis, nutritional interventions, sleep, stress, sedentary behavior, gut microbiota

## Abstract

This perspective article is a product of the work of an expert group within the Prebiotic Task Force convened by the International Life Sciences Institute Europe, a non-profit organization that brings together experts from academia, industry, and public service to catalyze nutrition science for public benefit. An expert group was conceived in October 2023 to discuss the evidence base on the use of prebiotics to promote cognitive functioning, with a focus on highlighting knowledge gaps and proposing a list of recommendations to guide this specific area of research forward. To address this, we evaluated existing systematic reviews and meta-analyses of human intervention studies that examine the effects of prebiotics on cognitive functioning. These are predominantly conducted in healthy participants under basal conditions and have, to date, revealed limited effects. In this perspective, we propose that prebiotics should be investigated as agents to promote cognitive resilience by testing their effects on cognitive performance under certain cognition-taxing factors that individuals encounter across their lifespan. These include stress, poor sleep outcomes, sedentary behavior, and unhealthy dietary patterns, all of which have been shown to be associated with altered microbiome and impact global cognition or specific cognitive domains. In addition, we recommend identifying vulnerable populations that are either subclinical or that struggle chronically or periodically with 1 or more cognition-taxing factors, to better uncover the boundary conditions for prebiotic effectiveness. By broadening the scope of research to include diverse populations and challenging conditions in daily life or experimental settings, we can expand our understanding of the role of prebiotics not only in cognitive health or impairment, but also as potential preventative agents that may promote cognitive resilience during aging and in response to various lifestyle-related challenges.

**Statement of Significance**

This perspective article proposes directing research efforts on prebiotics and cognition toward targeting cognitive resilience, whereby prebiotics can be investigated as a means to maintain cognition under taxing conditions like stress, poor sleep, sedentary behavior, and unhealthy dietary patterns that may be acute, periodic, or chronic. In randomized controlled trials, these cognition-taxing factors can be induced in experimental paradigms in otherwise healthy participants, or by identifying vulnerable populations whose cognition is impaired by such factors.

## Introduction

Optimal cognitive performance is key to the livelihood of the individual, educational attainment, and societal integration, which also bears significant consequences for economic growth and healthcare provisions [1,2]. The recent surge of interest in nutrition as a modifiable factor to help maintain adequate cognitive functioning across the lifespan prompted research into a myriad of nutritional interventions [[3], [4], [5]] to modulate various cognitive domains including attention, executive functioning, learning and memory, language, perceptual motor control, and social cognition [6]. The increasing recognition of the role of the gut microbiome in sustaining brain health via the microbiota-gut-brain axis [[7], [8], [9]] has further spurred interest in prebiotics as potential interventions to modulate human cognition. According to the International Scientific Association for Probiotics and Prebiotics, prebiotics are defined as “substrate[s] that [are] selectively utilized by host microorganisms conferring a health benefit” [10]. These can be found in fruits, vegetables, and legumes, and are enriched in foods such as yogurts and cereals, among others. To date, synthesis of the randomized controlled trials (RCTs) that investigated the efficacy of prebiotic interventions reveal weak effects on cognitive functioning at best [[11], [12], [13], [14], [15]]. We speculate that this is derived from employing prebiotics to enhance already optimal cognitive functioning (for example, testing effects predominantly in cognitively healthy participants). We propose that prebiotics should additionally be investigated as agents to promote cognitive resilience, defined as the “capacity to overcome the negative effects of setbacks and associated stress on cognitive function or performance” [16]. Consequently, this article advances the perspective of targeting cognitive resilience by first testing the effects of prebiotics on cognitive performance under certain cognition-taxing factors that individuals encounter acutely, periodically, or chronically across their lifespan, namely: stress, poor sleep outcomes, sedentary behavior, and unhealthy dietary patterns. Second, we recommend identifying vulnerable populations that are either subclinical or that struggle chronically with 1 or more of the above-mentioned cognition-taxing factors, to be included in RCTs that examine the effects of prebiotics on cognition. In what follows, we begin by briefly describing the current state of the evidence on prebiotics and cognition across the lifespan and then we highlight methodological limitations and caveats when interpreting the available findings. We proceed to describe the cognition-taxing factors under which prebiotic supplementation may promote cognitive resilience. Finally, we provide recommendations for future research in this area to overcome the current methodological limitations and address existing knowledge gaps. We developed these recommendations following guidance from European Food Safety Authority (EFSA) and Food and Drug Administration (FDA) on functional health claims and therefore, to establish the causal-relationship effects, we focus on healthy and subclinical populations and not on patients with clinically diagnosed conditions [17,18].

## Prebiotics and Cognition across the Lifespan: A Synopsis

Evidence within the field of the microbiota-gut-brain axis has revealed the important role of the gut microbiota in cognition, such that cognitive functions can be compromised after disruptions of the intestinal microbial community [19,20] and that interventions targeting the growth of beneficial gut bacteria can support, prevent a decline in, or restore cognitive functioning [12,21]. Intake of prebiotics is particularly interesting because these substrates are found in a diverse diet containing fruits, vegetables, legumes, and cereals and their beneficial effects on spatial learning ability [22], memory [[22], [23], [24], [25]], and reversal of cognitive deficits in Alzheimer's disease models [26] have been demonstrated in preclinical studies. Prebiotics primarily exert their influence on brain function through the production of short-chain fatty acids (SCFAs) after their fermentation by gut bacteria [27]. SCFAs can directly or indirectly impact the brain through the immune, endocrine, vagal, and other humoral pathways [27,28].

In contrast to the preclinical evidence base, there is a paucity of human clinical trials, with the existing literature showing both inconclusive evidence and knowledge gaps. For example, Desmedt et al. [14] identified significant, but selective, effects of chronic prebiotic interventions in healthy adults on immediate recall, recognition memory [29], and emotional vigilance [30], and greater, but inconsistent effects of acute interventions on recall, recognition [31], and executive function [14,32]. However, more recent systematic reviews [12] and meta-analyses in adults, children, and adolescents [13,15] concluded that insufficient evidence is available to confirm cognitive benefits after prebiotic interventions.

Although useful, the current systematic reviews and meta-analyses should be interpreted with some caveats in mind. The conclusions that can be drawn from synthesizing the current findings in humans are limited in their scope, because data are pooled across experimental studies that adopt heterogeneous methodologies and comprise small sample sizes. Some studies possess moderate to high risk of bias and are methodologically limited due to study designs and lack of adequate controls. Importantly, diverse prebiotics at different dosages have been administered for varying intervention periods (ranging from 10 min to 13 wk). The lack of consistent measurement of gut microbiota composition and resultant microbial metabolites as markers of prebiotic fermentation (for example, SCFAs) prevents identifying which prebiotics, at which dosages, and across which timeframes can exert a reliable and substantial effect on cognition. Similarly, the lack of multiple testing corrections when using cognitive testing batteries leads to an increase in rates of false positive findings. Finally, studies of the potential cognitive benefits of prebiotics require validated and sufficiently sensitive cognitive tests appropriate for the population of interest. The current use of a variety of measures hinders the ability to deduce whether the potential benefits of prebiotics could be attributable to an improvement in overall cognitive functioning or a specific domain of cognition (for example, memory or attention), further limiting the conclusions that can be drawn from systematic reviews and meta-analyses.

## Cognitive Resilience: A Missing Window of Opportunity to Reveal Prebiotic Efficacy beyond Health and Disease

**FIGURE 1:**
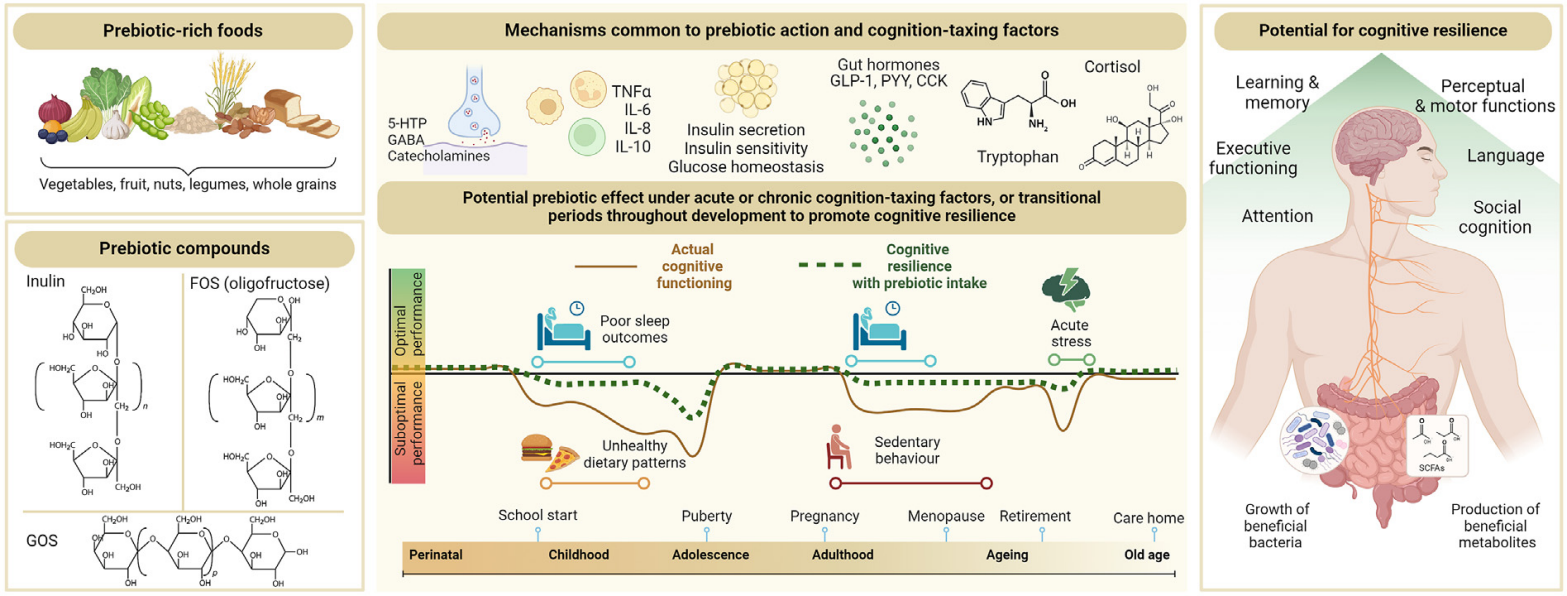
Potential effects of prebiotics on cognitive resilience. Prebiotic-rich foods and prebiotic compounds such as inulin, FOS, and GOS should be investigated in healthy individuals exposed to cognition-taxing factors or to target populations where cognition is acutely or chronically compromised. Cognition-taxing factors may occur across the lifespan and include exposure to stress, poor sleep outcomes, sedentary behavior, or unhealthy dietary patterns. These factors can be acutely present in daily life or evoked in experimental settings, or can be chronically present in specific samples such as individuals with habitual reduced intake of dietary fiber or low levels of physical exercise. The effects of prebiotics can be studied under exposure to one or more cognition-taxing factors, on one or more cognitive domains that are most impacted by such factors. In addition, the impact of these cognition-taxing factors and the potential rescuing effects of prebiotics may differ depending on the target developmental epoch, such as school start and puberty, or later in the lifespan due to menopause and retirement. Various mechanisms are shared between the effects of prebiotics on host physiology and the effects of cognition-taxing factors on cognitive performance, including modulation of neuroendocrine factors and host metabolism. By fostering the growth of beneficial bacteria and increasing the production of SCFAs, prebiotics may have the potential to promote cognitive resilience across various cognitive domains. 5-HTP, 5-hydroxytryptophan; CCK, cholecystokinin; FOS, fructooligosaccharides; GABA, gamma-aminobutyric acid; GLP-1, glucagon-like peptide 1; GOS, galactooligosaccharides; PYY, peptide tyrosine tyrosine; SCFA, short-chain fatty acid. Created in BioRender.com.

The existing evidence base comprises predominantly cognitively healthy adult participants [12,14,15]. Specifically, of the 8 prebiotic studies reported by Desmedt et al. [14] and the 5 prebiotic studies reported by Marx et al. [15], 7 and 3 studies were conducted in healthy adults, respectively. This, in turn, does not rule out the possibility that the weak and meta-analytically inconclusive findings may be driven by a ceiling effect on cognitive assessments. This ceiling effect might indicate that prebiotics do not exert additional beneficial effects in already cognitively “healthy” individuals. In our view, the current evidence base would benefit substantially from investigating the effects of prebiotics on cognitive resilience (Figure 1). As mentioned above, cognitive resilience refers to the ability of the individual to maintain or regain cognitive functioning under stress or other challenging situations [16]. Rather than assessing cognition in healthy participants under “non-demanding” conditions, where a ceiling effect is easily reached, researchers could emulate compromised cognition in the laboratory by administering certain challenges known to impair cognition or by targeting certain populations with suboptimal cognitive functioning. Such populations could be older adults with subjective cognitive decline or prodromal Alzheimer’s disease, in which symptoms are not severe enough to interfere with daily functioning or to meet the criteria for dementia diagnosis. In other words, prebiotics could show substantial benefit to cognition only under scenarios where it is transiently or chronically (yet subclinically) suboptimal.

Here we propose a novel angle to investigate the potential of prebiotic effects on cognition. Specifically, targeting suboptimal cognition in healthy individuals that may arise due to stress, poor sleep outcomes, sedentary behavior characterized by little physical exercise, and unhealthy dietary patterns characterized by reduced dietary fiber intake, may provide a window of opportunity for prebiotics to maintain optimal cognitive functioning (Figure 1). These “cognition-taxing factors” are described below with regard to their mode of action, associations with gut microbiota alterations, effects on specific cognitive domains, and where possible, the available preclinical studies that administered prebiotics under these conditions to promote cognitive resilience are discussed.

### Stress

Stress is a response to a challenge of an uncontrollable and unpredictable nature that exceeds current coping resources, resulting in the activation of the sympathetic adrenal medullary system and the hypothalamic-pituitary-adrenal axis, and the subsequent release of stress hormones. Stress impacts gut microbiota by altering its diversity and composition, with different effects observed in acute compared with chronic stress. Acute stress can influence the gut microbiome by interacting with stress hormones and may be mitigated by probiotics, although real-time sample collection poses challenges [33]. Chronic stress leads to inconsistent effects on alpha diversity, increases microbial volatility, and often reduces beneficial bacteria like *Lactobacillus*, *Bifidobacterium*, and *Akkermansia* while enriching pathogenic bacteria such as *Escherichia-Shigella* [33]. However, there is currently no consensus on which microbial taxa are consistently modified by prolonged or chronic stress. Perceived stress in humans has been linked to decreased microbial diversity but an increase in immunomodulatory microbes like *Bacteroides*, *Streptococcus*, and *Veillonella*, potentially helping maintain gut health despite stress [34].

Stress impairs executive functions, particularly working memory and cognitive flexibility [[35], [36], [37]], possibly by biasing cognitive resources toward dealing with the current stressor, thus limiting available resources for other cognitive processes [38]. However, acute stress might also enhance certain cognitive aspects, including response inhibition, memory encoding, and retention [37]. Hence, examining a predefined cognitive domain under different stress conditions and after prebiotic administration is especially important [39].

Preclinical evidence on the effects of prebiotics on stress-impaired cognition is rather limited. Burokas et al. [40] showed no effects of fructooligosaccharides (FOS) and galactooligosaccharides (GOS) supplementation on recognition memory under chronic stress. Interestingly, another study evaluated the effects of FOS-inulin supplementation on stress-induced impairment in social cognition in aged mice. Although in this study stress did not exacerbate social novelty deficits, FOS-inulin improved overall social recognition in aged mice, suggesting that a prebiotic dietary intervention in aging can mitigate age-dependent behavioral deficits [41].

### Poor sleep outcomes

Poor sleep outcomes (that is, too little, or too much sleep or low sleep quality) exhibit significant deleterious effects on cognitive performance across most domains [[42], [43], [44]]. Poor sleep outcomes trigger a cascade of physiological changes, affecting neurotransmitter balance [45], multiple endocrine alterations [46,47], inflammation [48], and oxidative stress [47,48]. Sleep quality and patterns further have significant effects on gut microbiota composition and diversity. Poor sleep efficiency and greater variability in sleep duration are associated with reduced gut microbiome richness and diversity and alterations in beta-diversity, indicating that consistent, high-quality sleep supports a more diverse and stable gut microbiome [49]. Subjective poor sleep quality is marginally linked to lower alpha diversity in older men, although this association can lose significance after adjusting for other variables [50]. Additionally, a higher relative abundance of taxa such as *F. prausnitzii*, *P. copri*, *B. vulgatus*, *B. dorei*, *A. onderdonkii*, and *R. bicirculans* is associated with better sleep quality and regularity, with the first 4 bacteria known to produce butyrate [49]. Furthermore, a diverse gut microbiome was positively correlated with better sleep efficiency and total sleep time, and negatively correlated with sleep fragmentation, suggesting that a diverse gut microbiome promotes healthier sleep [51].

Sleep has negative consequences for overall cognitive function, particularly attention, working memory, executive functioning, and learning and memory consolidation [52]. Interestingly, 1 study found a significant positive correlation between microbiome richness and both sleep efficiency and abstract matching, which measures the abstraction and flexibility components of executive function [51]. The significant effects of poor sleep outcomes on cognitive functioning during key life stages such as childhood, adolescence, and menopause have been highlighted in the literature [53,54]. Yet little research to date has systematically assessed the role of dietary interventions on the relationship between sleep and cognition during these life stages [52]. Interestingly, one preclinical study investigated the effects of individual and combined effects of sleep disruption and social stress on object location memory after control or prebiotic diet (GOS and polydextrose) [55]. When undergoing sleep disruption alone, both prebiotic- and control-fed animals exhibited comparable object location memory retention indices. However, prebiotics rescued the effects on memory when the animals were subjected to both sleep disruption and social stress [55].

### Sedentary behavior

Lack of physical exercise leads to metabolic disruptions that hinder neural growth factor release, energy supply to the brain, anti-inflammatory cytokine release, and neurotransmitter production [56], subsequently posing a risk for cognitive decline and dementia [57]. Sedentary behavior is additionally associated with lower microbial diversity and an unfavorable gut microbiota composition, including higher levels of *Escherichia coli* and reduced capacity for carbohydrate degradation [58,59]. In contrast, exercise positively impacts gut microbiota by increasing microbial diversity and promoting growth of beneficial bacteria, such as *Faecalibacterium prausnitzii* and *Roseburia spp.*, *Veillonella* and *Akkermansia*, which are known to produce SCFAs [58,60,61]. Athletes exhibit greater gut microbial diversity and higher relative abundance of beneficial bacterial taxa compared with sedentary individuals [60,61]. However, the specific effects of exercise on gut microbiota may vary depending on factors like diet, exercise intensity, duration, and individual BMI, necessitating further longitudinal and experimental studies to fully understand these relationships [58,60,61].

Systematic reviews highlight a relationship between increased sedentary behavior and poor global cognitive function and processing speed [62,63], and that cognitively impaired populations (that is, those diagnosed with mild cognitive impairment or dementia) tend to spend more time sedentary than cognitively healthy individuals [63]. Furthermore, in a recent large systematic review and meta-analysis, physical activity was associated with better late-life cognition, particularly in relation to episodic memory and verbal fluency [64].

### Unhealthy dietary patterns

Unhealthy dietary patterns—high in saturated fats, sugar, and low in plant-based foods—are linked to a higher risk of dementia [65] and poorer cognitive function in children and adolescents [66]. Such dietary patterns are further associated with detrimental effects on the gut microbiota, including lower richness and diversity and an unhealthy metabolic state (insulin resistance, body fat %, BMI, triglycerides, lipoproteins) [67]. Obesity and type 2 diabetes mellitus (T2DM) are linked to altered gut microbiota composition and decreased diversity, with a higher Firmicutes-to-Bacteroidetes ratio and decreased microbial gene richness [68]. Moreover, decreased abundance of butyrate-producing bacteria such as *Faecalibacterium prausnitzii* and *Roseburia spp*. was observed in T2DM and metabolically compromised individuals. Additionally, Western diets low in dietary fiber lead to proteolytic fermentation, producing compounds that negatively impact gut and metabolic health [68]. Conversely, diets rich in PUFAs do not seem to negatively affect gut microbiota or metabolic health outcomes [67].

Such unhealthy dietary patterns impact cognitive functions, particularly verbal learning and memory [69] and executive functioning [70] due to increased inflammation, oxidative stress, and promotion of insulin resistance [71]. In a preclinical study, Shi et al. [72] observed impaired cognition in a mouse model deprived of dietary fiber for 15 wk, mediated by alterations in SCFA production and inflammation across the microbiota-hippocampal axis. Interestingly, prebiotic candidate xylooligosaccharides treatment was found to reverse the deleterious effects of chronic exposure to a high-fat diet on spatial learning and memory [73].

**FIGURE 2:**
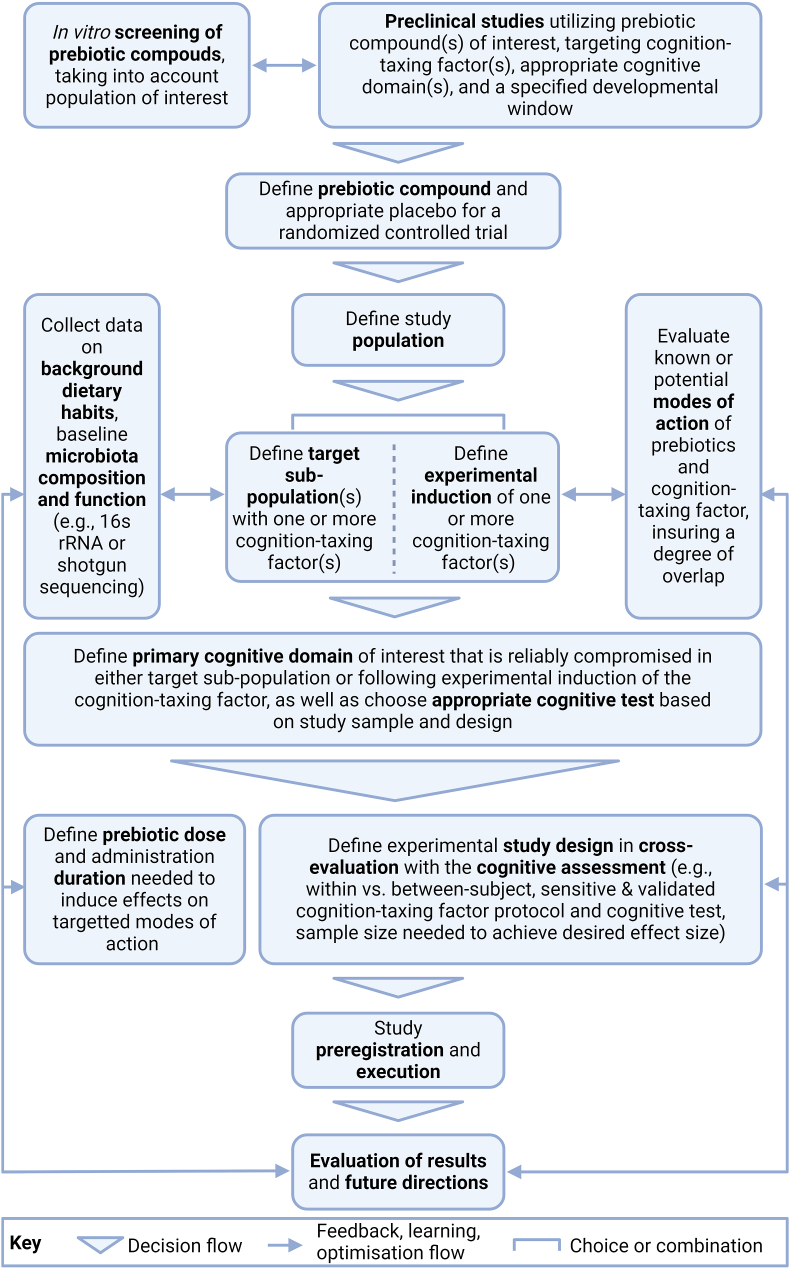
A roadmap to conduct randomized placebo-controlled trials targeting cognitive resilience.

The mechanisms by which the above-mentioned behaviors tax cognition largely align with the pathways through which prebiotics can modulate brain function (Figure 1). By promoting the release of SCFAs, prebiotics may exhibit anti-inflammatory effects, strengthen intestinal and blood-brain barriers, improve glycaemic control, and reduce stress reactivity, potentially restoring or rescuing cognitive performance impaired by stress, poor sleep outcomes, sedentary behavior, and unhealthy dietary patterns [28,74]. The emerging evidence on the gut microbiota alterations associated with these cognition-taxing factors further underscores the potential of intervening at the level of the microbiota using prebiotics to promote cognition. Future studies should ensure adequate measurement of the composition and function of the gut microbiota, and associated potential mechanisms of action (see Table 1 and Figure 2) to better understand and substantiate the conditions under which prebiotics are effective.

**TABLE 1:** Overview of techniques and biomarkers to utilize or measure in prebiotic-cognition studies.

Direct microbial markers	
Faucal microbiota (e.g., composition, metabolomics, transcriptomics)	An indicator of the microbial diversity and microbial function within the colon
Serum microbial components (e.g., microbial DNA, lipopolysaccharides)	Markers of gut barrier integrity and its resistance to microbial (product) translocation into the blood
Circulating microbe-derived/influenced factors	
Microbial metabolites (e.g., SCFAs, methylamines, indoles, tryptophan metabolites)	The products of microbial prebiotic metabolism can be sampled from both faces and plasma, and assayed by, for example, ^1^H-nuclear magnetic resonance or mass spectrometry. Additionally, innovative isotope labeling techniques can be used to quantify production of colonic prebiotic-derived active products
Diet-derived factors (e.g., polyphenols, lipids/phospholipids, micro/macronutrients)	Indicative of dietary intake
Host metabolomics (e.g., amino acids, sugars, fatty acids, lipids, neurotransmitters, and steroids)	Measures of host molecules influenced by microbial activity, assayed by, e.g., ^1^H-nuclear magnetic resonance or mass spectrometry
Inflammatory markers (e.g., leukocyte phenotypes, pro- and anti-inflammatory cytokines, chemokines, erythrocyte sedimentation rate, C-reactive protein, serum amyloid A, faucal calprotectin)	Measures of immune system activity
Brain-derived proteins (e.g., GFAP, S100β, NfL, brain-derived neurotrophic factor)	Markers of cerebrovascular integrity and/or indicators of brain function. Also widely used to assess the extent of brain injury

Neuroimaging, other neural and psychophysiological markers	

Structural imaging [e.g., structural MRI, Diffusion tensor imaging (structural connectivity)]	Provides high spatial resolution and soft tissue contrasts to measure brain morphometry (e.g., grey matter volume, cortical thickness) or assesses the microstructure of white matter and anatomical connectivity and integrity
Functional imaging (e.g., functional MRI, resting state functional connectivity, positron emission tomography, arterial spin labeling)	Measures brain activity by detecting changes in blood oxygenation and flow during rest or an evoked task or assesses the temporal correlation of the low frequency fluctuations between different brain regions
(Proton) Magnetic resonance spectroscopy	Measures concentrations of metabolites and neurotransmitters in the brain (e.g., glutamate, gamma-aminobutyric acid)
Other neural markers (e.g., electroencephalogram, functional near-infrared spectroscopy, magnetoencephalography)	Measures cerebral electrical activity, hemodynamic responses, magnetic fields produced by electrical activity
Other psychophysiological measures (e.g., blood pressure, heart rate (variability), respiration, electrodermal activity, pupillometry etc.)	

Abbreviations: GFAP, glial fibrillary acidic protein; NfL, neurofilament light protein; SCFA, short-chain fatty acid.

## A Focus on Experimental Conditions and Target Populations to Unveil Potential Prebiotic Effects on Cognition

Examining whether prebiotics can promote cognitive resilience under the above-mentioned cognition-taxing factors can be accomplished either within controlled experimental settings—where 1 or more cognition-taxing factor is evoked—or by studying target populations—where 1 or more cognition-taxing factor is already periodically or chronically present. For example, stress can be evoked acutely in human participants in the laboratory using various experimental paradigms [75,76], or otherwise, healthy participants can be selected based on their (sub)chronic exposure to stress (for example, examination periods) or based on reports of chronic stress or belongingness to a vulnerable, chronically stressed subgroup (for example, caregiver of a family member with a terminal illness, emergency room physician, medical students). Similarly, poor sleep outcomes can also be acutely evoked in the laboratory [77], or alternatively, otherwise healthy participants whose sleep schedule is often disrupted as is the case in (night-) shift workers such as truck drivers, flight attendants, security personnel, or doctors, can be preselected [78]. The remaining cognition-taxing factors, namely adopting sedentary behavior or unhealthy dietary patterns, are more challenging to induce (semi)acutely in controlled laboratory settings or evoke for long enough periods to first ensure a measurable compromised cognitive functioning before prebiotic intervention. Nevertheless, subgroups could be selected based on their reports of exercise frequency and detailed food diary records.

It should be noted that cognition-taxing factors may vary in their magnitude of effect on cognitive performance—possibly leading to diverse responses to prebiotic supplementation—depending on the developmental stage of the individual (Figure 1). For instance, the impact of sleep deprivation or acute stress on cognition may be particularly pronounced during critical developmental windows [79], concomitant with ongoing maturation and increased neuronal plasticity, which in turn may lead to atypical behavioral patterns and abnormal brain network maturation [80,81]. Hence, it is imperative to systematically investigate the utility of prebiotic interventions across distinct developmental epochs. Although the significance of healthy nutrition and dietary interventions during the early pre- and postnatal years is firmly established [13], little evidence is available for later years, notwithstanding animal research highlighting critical windows in late childhood and adolescence [82,83]. Consequently, future investigations are now needed to establish the efficacy of prebiotic interventions in supporting cognitive functioning during key transitional stages across the lifespan that occur during childhood and adolescence, such as beginning formal education, and the transition into secondary school and university, with or without the presence of cognition-taxing factors. Adopting a lifespan perspective, such approaches could also extend into later life transitions, such as menopause [84], retirement [85], and old age, even in ostensibly healthy individuals. In line with this, a recent study demonstrated that 12-wk prebiotic inulin:oligofructose intake in healthy older twins (age >60) resulted in some improvements in cognition, particularly in relation to associative learning and memory [86].

## Recommendation for Future Research on Prebiotics and Cognition

Given our current perspective on the field of prebiotics and cognition, we propose the following recommendations to progress this area of research. First, assessing the effects of prebiotics in healthy participants across the lifespan either under cognition-taxing challenges or due to cognition-taxing chronic conditions may help better characterize the potential of prebiotics to benefit cognition, particularly in defining windows of opportunity for intervention, as well as better estimating their magnitude of effect (Figure 2). It may be further advantageous to test the effects of prebiotics under the combined effect of stress, poor sleep outcomes, sedentary behaviors, and unhealthy dietary patterns [87]. This can be done using the integrated 24-h time-use paradigm [88] or by applying multiple experimental paradigms in a controlled laboratory setting. This recommendation is in addition to investigating the efficacy of prebiotics in vulnerable populations with subclinical manifestations of cognition-impairing conditions such as mild cognitive impairment or early dementia [21], chemotherapy-induced cognitive impairment and post-operative cognitive decline, mood-related disorders, individuals with subjective cognitive decline, and individuals undergoing key transitional periods in life (for example, puberty, menopause, retirement). Indeed, some ongoing studies at present are assessing the effects of prebiotics on cognition in individuals with subjective cognitive decline who may additionally exhibit high adherence to unhealthy dietary patterns [89], or in healthy individuals undergoing acute psychosocial stress in the laboratory [90], or sleep restriction and circadian misalignment [78]. Furthermore, when extending this line of investigation to vulnerable populations that fall outside healthy and subclinical populations classified within EFSA and FDA guidelines for functional health claims, it will be important to consider whether prebiotics can help protect against the detrimental effects of commonly used drugs on cognitive functioning, or even maximize their therapeutic efficacy [91]. Various drugs including antibiotics, statins, metformin, nonsteroidal anti-inflammatory drugs, proton-pump inhibitors (PPIs), antidepressants, benzodiazepines, antipsychotics, opioids, and antihistamines can have dual effects on the gut microbiome as well as cognition [[92], [93], [94]]. Daily prescribed drugs are suggested to induce dysbiosis in addition to their intended pharmacological effects [95], and recent evidence suggests that when controlling for the use of multiple drugs, PPIs, metformin, antibiotics, and laxatives reveal the strongest associations with the composition and metabolic function of the gut microbiota [96]. Importantly, commonly prescribed drugs are known to impair cognition as a side effect, particularly after long-term use [97], which is especially disconcerting when considering polypharmacy in elderly populations [98]. However, the majority of studies recruit individuals who do not take prescribed drugs, limiting the possibility of gaining more insight into the complex and bidirectional interaction between the gut microbiome and effects of commonly used drugs. Emerging research is investigating the intertwined effects of prebiotics, drug therapies, and cognition and whether prebiotics may have the potential to counteract the reduced cognitive functioning caused by those drugs. For example, co-administration of prebiotics to rats receiving olanzapine may influence cognitive outcomes via the modulation of N-methyl-D-aspartate (NMDA) receptor function and optimization of drug efficacy [99]. Future research into the bidirectional interactions between drug intake and the gut microbiome is needed to provide insight into pharmacomicrobiomic interactions and must be considered an area of significant interest to fully explore the potential of prebiotics in protecting cognitive function [91].

Second, it is imperative to note that the choice of cognitive domain of interest should be guided by the choice of the cognition-taxing factor and the relevant study population. In addition, the reader is referred to existing guiding documents to consider the cognitive domains and tests most sensitive to nutritional interventions [100,101]. It will be essential to adopt co-production approaches for these intervention protocols where possible, to ensure that participants’ needs and difficulties can be adequately addressed. This will also further ensure adherence and the successful implementation of the prebiotic intervention.

Finally, it is important to assess relevant biomarkers and potential mechanisms of action, including immune functioning, neuroendocrine and neural readouts, and the effect on specific microbial metabolites to facilitate the identification of successful prebiotic interventions in terms of type, dosage, and duration of administration (see Table 1 for suggested relevant biomarkers and measures). In parallel, incorporating the characterization of the gut microbiota of a given sample may be important to define subgroups to successfully account for some of the variabilities impacting cognitive outcomes. This can be done by leveraging standardized and state of the art gut microbiome analysis methodologies (for example, shotgun sequencing) to understand the impact of prebiotics in intervention studies, with concurrent in vitro investigations to gain a deeper understanding of the mechanisms of action. On the other hand, novel in vitro and in silico methodologies could be developed to screen for innovative solutions targeting cognition. Taken together, employing these approaches should help in revealing whether prebiotics can act as acutely rescuing agents under cognition-taxing conditions, or whether their prolonged daily intake is required to promote potential protective effects or foster cognitive resilience in humans.

## Author contributions

The authors’ responsibilities were as follows – BD, AC, VD, YCZ: conceptualized the manuscript; BD, NBB, HS: drafted the initial subsections of the manuscript, with further input from FTR, KCK, AC, VD, FR, GFD, SM, RJB, YV, YCZ, PH, SM and BD; BD: edited the manuscript; and all authors: read and approved the final manuscript.

## Conflict of interest

A. Chakrabarti is an employee of Cargill Inc., F. Respondek is an employee of CP Kelco, G. Dodd is an employee of Clasado Biosciences, V. Dam is an employee of Sensus B.V. and Y. C. Zanzer is an employee of BENEO Institute/Südzucker Group. Other authors have no competing interests.
